# Arnold-Chiari Malformation and Scoliosis: A Chronic Lung Collapse Mimicking Sepsis

**DOI:** 10.7759/cureus.15292

**Published:** 2021-05-28

**Authors:** Francisco J Somoza-Cano, Abdul Rahman Al Armashi, Faris Hammad, Kanchi Patell, Keyvan Ravakhah

**Affiliations:** 1 Internal Medicine, St. Vincent Charity Medical Center, Cleveland, USA

**Keywords:** arnold-chiari malformation, scoliosis, neuromuscular scoliosis, sepsis, chiari type 2 malformation

## Abstract

Scoliosis is a deformity of the spine caused by excessive lateral curvature. Compared to other variants, neuromuscular scoliosis is more likely to progress, altering the body’s normal architecture in a relatively short period of time. Furthermore, patients with Arnold-Chiari malformation or Chiari malformation (CM) type 2 have intrinsic neurological complications that might entangle the initial clinical assessment. A 24-year-old woman with a history of scoliosis and CM type 2 status post-tracheostomy was admitted from a skilled nursing facility after a one-day history of low blood pressure, leukocytosis, and an outpatient chest X-ray suspicious for pneumonia. Physical examination was remarkable for hypotension and decreased breath sounds at the left pulmonary base. A tracheostomy tube and central venous catheter were noticed. Initial laboratory results revealed leukocytosis with borderline bandemia and a chest X-ray with a left lower lung consolidation. She was treated as a case of sepsis, for which broad-spectrum antibiotics were immediately started. However, upon review of charts, the patient’s objective findings were similar to a previous admission. Chest computed tomography scan revealed atelectasis in the left lower lung with no signs of consolidation, effusions, or abscesses. After extensive workup, no identifiable cause was found to suggest an acute process. Antibiotic therapy was halted and the patient was discharged to her nursing home. This case presents a patient with CM type 2 and scoliosis complicated by chronic and worsening atelectasis. Accurate initial assessment and communication between providers are paramount to avoid overtreatment.

## Introduction

Scoliosis is a common malformation of the spine that affects up to nine million people in the United States alone [1]. In neuromuscular scoliosis, an underlying pathology alters the trunk’s muscle tonicity, inducing a disharmonious control of the musculature around the spinal axis that progressively worsens [2]. Moreover, Arnold-Chiari or Chiari malformation (CM) type 2 is a congenital neuromuscular disorder that causes herniation of the brainstem, cerebellar tonsils, and vermis. This malformation is frequently accompanied by spina bifida, increasing the degree of neurological compromise. CM type II has a 3% neonatal in-hospital mortality and a 15% three-year mortality rate. Those who survive have worsening motor dysfunction over time to the point of respiratory failure [3]. Moreover, scoliosis affects the movement of the chest wall during inspiration and expiration. It directly affects the chest wall compliance and indirectly affects lung compliance by decreasing ventilation with resulting atelectasis. If scoliosis is left untreated, pulmonary hypertension and chronic respiratory failure may develop, causing a significant portion of the morbimortality in these patients [4].

## Case presentation

A 24-year-old woman with a past medical history of CM type 2 status post-tracheostomy, scoliosis, spina bifida repaired at birth, and hydrocephalus status post-ventriculoperitoneal shunt was directly admitted to the Intensive Care Unit from a nursing home after a one-day history of low blood pressure and leukocytosis. Outpatient chest X-ray showed a possible consolidation on the left pulmonary base. On admission, the patient was alert, following simple commands, and nonverbal, which she had been since childhood. Physical examination was remarkable for short stature (116 cm) and scoliotic posture. A right central venous catheter (CVC) and a tracheostomy tube were also noted. Blood pressure was 84/53 mmHg, temperature 36.6°C, heart rate 88/minute, and respiratory rate was 24/minute. Reduced breath sounds were heard over the left pulmonary base. Her initial workup was remarkable for a total leucocyte count of 12.4 K/uL (reference range: 3.9-11 K/uL) with 10% bands (reference range: 0-5%). Electrolytes were within normal limits. Creatinine was 0.19 mg/dL (reference range: 0.3-1.5 mg/dL) and blood urea nitrogen was 3 mg/dL (reference range: 5-24 mg/dL). Lactic acid was 0.8 mmol/L (reference range: 0.8-2.0 mmol/L), and inflammatory markers showed a C-reactive protein of 50.2 mg/L (reference range: 0-3 mg/L), erythrocyte sedimentation rate of 98 mm/hour (reference range: 0-20 mm/hour), and a procalcitonin of 0.05 ng/mL (reference range: less than 0.05 ng/mL). Chest X-ray revealed severe S-shaped scoliosis of the thoracolumbar spine and a left basilar airspace consolidation.

**Figure 1:**
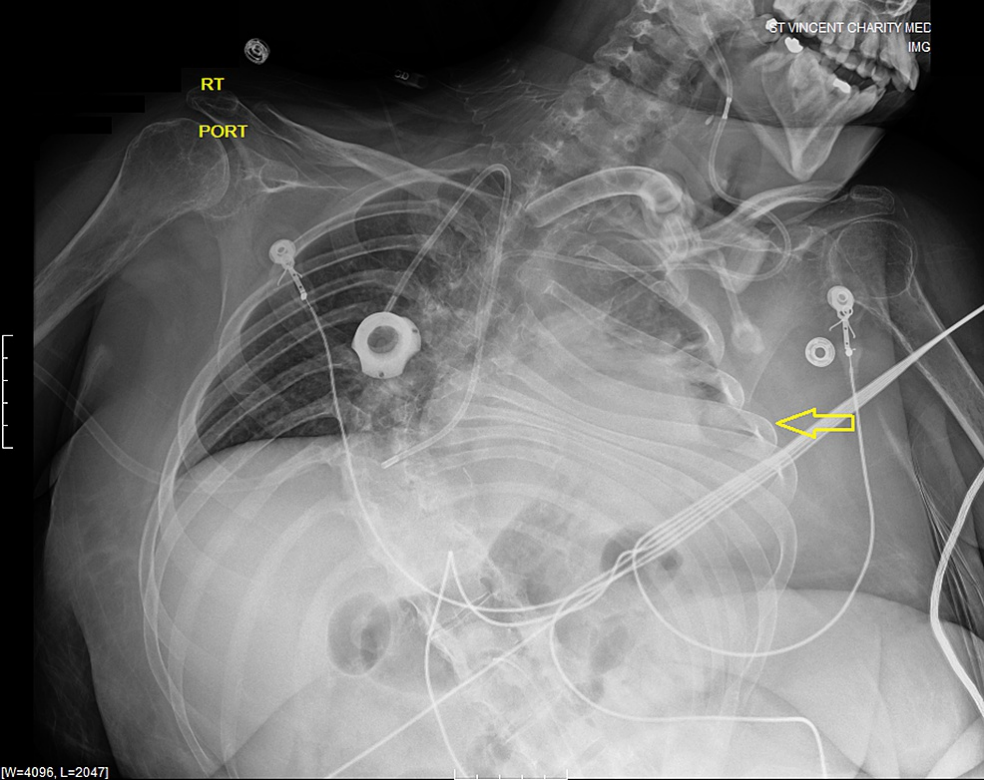
Chest X-ray. A single anteroposterior portable radiograph of the chest was obtained on admission. It showed a right internal jugular CVC with the tip overlying the cavoatrial junction, evidence of a tracheostomy tube present in situ, and a shunt catheter over the chest. Severe S-shaped scoliosis of the thoracolumbar spine and dense airspace consolidation over the left lung base was noted. CVC: central venous catheter

Broad-spectrum antibiotics and fluid resuscitation were immediately started. However, further review of past admissions revealed a persistent borderline low blood pressure, chronic leukocytosis, and a chest X-ray with atelectasis on the left lower base. A chest computed tomography (CT) scan showed a marked elevation of the left hemidiaphragm associated with the known scoliosis. Consequently, a resultant volume loss in the left hemithorax was noted with compressive atelectasis in the left lower lobe accounting for the radiographic findings.

**Figure 2:**
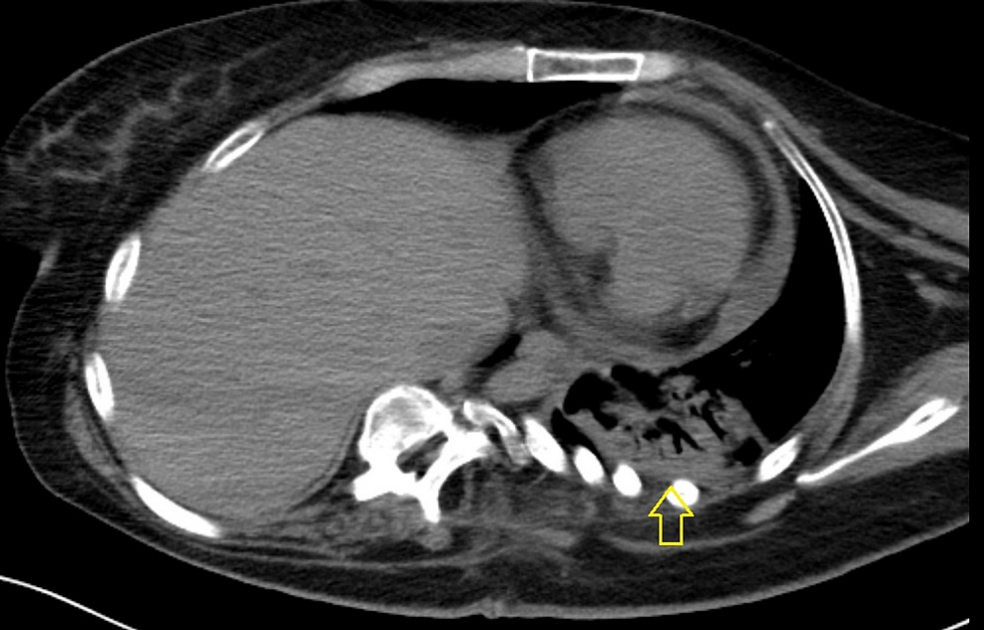
Chest CT scan. A considerable elevation of the left hemidiaphragm with severe dextroconvex scoliosis of the lower thoracic and upper lumbar spine was reported. These findings contributed to severe volume loss in the left hemithorax. Finally, there was compressive atelectasis in the left lower lobe with no evidence of pleural effusion. CT: computed tomography

Blood cultures were negative after three days and the patient was discharged from the hospital. The nursing home was advised on the findings to prevent similar readmissions to other facilities.

## Discussion

Scoliosis is a spinal deformity consisting of lateral curvature and rotation of the vertebrae. It is more commonly diagnosed during childhood and is classified as neuromuscular, congenital, syndrome-related, secondary to a specific pathology, and idiopathic. Its usually benign clinical course may be complicated by severity or when various neuromuscular conditions coexist [5,6].

Moreover, CMs are a group of deformities of the posterior fossa and hindbrain that range from mild cerebellar tonsillar herniation to the absence of the cerebellum. These mutations are commonly associated with other neurological defects such as hydrocephalus, syrinx, or spinal dysraphism [3]. There are multiple proposed theories for the etiology of these syndromes with the likelihood that different mechanisms lead to similar results [7]. CM type 1 is the most common type and occurs in approximately 0.5-3.5% of the general population or 1/1000 births [8]. CM type 2 is more infrequent, occurring in 0.44 every 1000 births, and is associated with neural tube defects in about 100% of cases [3]. Furthermore, the progressive neurodegeneration inevitably causes the arrest of normal stature development explaining chronic low blood pressure readings in these patients [9]. The main goal in these conditions is prevention with folate replacement therapy during the early stages of pregnancy.

Scoliosis is most frequently associated with CM type 1; however, CM type 2 is commonly associated with one or more syndromes associated with CM type 1 [10]. This clinical entity is not exceedingly rare in practice; nevertheless, there is a relative paucity of published research regarding spinal deformities associated with CMs. Thus, the pathophysiology of the spinal abnormalities and the long-term effects of the malformations remain poorly understood [11]. Nonetheless, the literature regarding the association of CM and scoliosis has been growing over the past three decades; however, no consensus on optimal management has been reached [12].

Furthermore, the current guidelines for sepsis specify the importance of starting empiric antibiotics within an hour when there is high suspicion for this syndrome [13]. In this patient, the possible consolidation was not the only finding that could cause septicemia. Indwelling catheters and tracheostomy tubes pose a critical risk for infection. Bacterial endocarditis, urinary tract infection, and meningitis are some of the conditions associated with the types of catheters our patient had. Therefore, the initial decision to treat the patient with antibiotics was appropriate. However, potential confounding variables may lead to overtreatment. In a patient with CM type 2 and scoliosis, the chronic and continuously worsening spinal deformity has a great impact on the normal architecture of internal organs. Scoliosis decreases the chest wall compliance indirectly due to progressive atelectasis and air-trapping. Consequently, a significant increase in the work of breathing must be produced to maintain adequate oxygenation explaining the elevated respiratory rate. The associated respiratory muscle weakness inevitably leads to chronic respiratory failure, as seen in our patient [13]. Moreover, long-standing catheters may cause chronic inflammation with elevated inflammatory markers [14]. Appropriate documentation of findings and interinstitutional communication is of the utmost importance to ensure adequate early assessment.

## Conclusions

The potential drawbacks of scoliosis complicated with an incapacitating neurological disorder must be acknowledged for prompt and accurate diagnosis of the present illness. It may prove challenging to distinguish between an acute process that needs immediate attention and a benign situation that can be managed with conservative therapy. Confounders might cause overtreatment with its intrinsic detrimental side effects. Accurate clinical assessment and communication between medical providers are paramount to avoid unnecessary hospitalizations.
